# Vijana Vijiweni II: a cluster-randomized trial to evaluate the efficacy of a microfinance and peer health leadership intervention for HIV and intimate partner violence prevention among social networks of young men in Dar es Salaam

**DOI:** 10.1186/s12889-016-2774-x

**Published:** 2016-02-03

**Authors:** Lusajo Kajula, Peter Balvanz, Mrema Noel Kilonzo, Gema Mwikoko, Thespina Yamanis, Marta Mulawa, Deus Kajuna, Lauren Hill, Donaldson Conserve, Heathe Luz McNaughton Reyes, Sheila Leatherman, Basant Singh, Suzanne Maman

**Affiliations:** 1 Department of Psychiatry and Mental Health, Muhimbili University of Health and Allied Sciences, PO Box 65466, Dar es Salaam, Tanzania; 2 Department of Health Behavior, The University of North Carolina at Chapel Hill, Gillings School of Global Public Health, Rosenau Hall, CB 7440, Chapel Hill, NC 27599 USA; 3 American University, School of International Service, 4400 Massachusetts Avenue, NW Washington, DC 20016, USA; 4 Department of Health Policy and Management, The University of North Carolina at Chapel Hill, Gillings School of Global Public Health, Rosenau Hall, CB 7411, Chapel Hill, NC 27599 USA; 5 Department of Psychiatry and Behavioral Sciences, Medical University of South Carolina, 67 President Street, Charleston, SC 29425 USA

**Keywords:** HIV, Intimate partner violence, Social networks, Intervention, Men, Tanzania

## Abstract

**Background:**

Intimate partner violence (IPV) and sexually transmitted infections (STIs), including HIV, remain important public health problems with devastating health effects for men and women in sub-Saharan Africa. There have been calls to engage men in prevention efforts, however, we lack effective approaches to reach and engage them. Social network approaches have demonstrated effective and sustained outcomes on changing risk behaviors in the U.S. Our team has identified and engaged naturally occurring social networks comprised mostly of young men in Dar es Salaam in an intervention designed to jointly reduce STI incidence and the perpetration of IPV. These stable networks are locally referred to as “camps.” In a pilot study we demonstrated the feasibility and acceptability of a combined microfinance and peer health leadership intervention within these camp-based peer networks.

**Methods design:**

We are implementing a cluster-randomized trial to evaluate the efficacy of an intervention combining microfinance with health leadership training in 60 camps in Dar es Salaam, Tanzania. Half of the camps have been randomized to the intervention arm, and half to a control arm. The camps in the intervention arm will receive a combined microfinance and health leadership intervention for a period of two years. The camps in the control arm will receive a delayed intervention. We have enrolled 1,258 men across the 60 study camps. Behavioral surveys will be conducted at baseline, 12-months post intervention launch and 30-month post intervention launch and biological samples will be drawn to test for Neisseria gonorrhea (NG), Chlamydia trachomatis (CT), and Trichomonas vaginalis (TV) at baseline and 30-months. The primary endpoints for assessing intervention impact are IPV perpetration and STI incidence.

**Discussion:**

This is the first cluster-randomized trial targeting social networks of men in sub-Saharan Africa that jointly addresses HIV and IPV perpetration and has both biological and behavioral endpoints. Effective approaches to engage men in HIV and IPV prevention are needed in low resource, high prevalence settings like Tanzania. If we determine that this approach is effective, we will examine how to adapt and scale up this approach to other urban, sub-Saharan African settings.

**Trial registration:**

Clinical Trials.gov: NCT01865383. Registration date: May 24, 2013.

## Background

Gender inequality is at the core of the HIV patterns that are evident in sub-Saharan African settings like Tanzania [1]. Research has documented that the power imbalance that exists between men and women are important drivers of women’s elevated HIV risk [2, 3]. Due to entrenched gender norms that provide men with the power and control in sexual relationships to determine the conditions, such as the timing, the level of protection, and the consensual nature of sex with their partners, men in sub-Saharan Africa are key targets for HIV prevention efforts [4–9]. Young men are particularly important targets because they are forming sexual partnership practices that become normative and consequently persist into adulthood [10].

Intimate partner violence (IPV) is a manifestation of the power imbalance that exists between men and women. The World Health Organization reports 30 % of ever-partnered women worldwide have experienced IPV in their life [11]. The Tanzanian DHS found that 31.8 % of women in Dar es Salaam reported experiencing physical violence in their lifetimes and 23.8 % of women in Dar es Salaam reported experiencing physical violence often or sometimes within the last 12 months [12]. The consequences of IPV for women are substantial and include mental health effects like incident depression, PTSD and suicidal ideation [13–16]; decreased use of contraceptives [17] and other reproductive health consequences [18]; elevated substance use [19]; increased risk for STIs including HIV [20, 21] and increased risk of chronic pain as well as non-fatal and fatal injuries [15, 17, 22]. IPV has also been associated with negative health outcomes for perpetrators including hazardous drinking, illicit drug use, mental health consequences, and elevated risk for STIs among men [8, 9, 23–28].

We continue to lack knowledge on how to access and engage young men at risk for HIV and IPV perpetration. Clinic based approaches may not be effective at reaching young men because young men access health services at disproportionately lower rates than females in part due to traditional masculine gender norms [29, 30]. Worksite and school based approaches may also not be the most effective approach, because many young men most at risk are not in school and not formally employed [31]. Intervention strategies to engage men are also lacking, particularly those that address the structural determinants of risk, such as poverty and unemployment. Unemployment has destructive physical and mental health effects on men [32], and the stress associated with men’s inability to meet their roles as economic providers for their sexual partners and their families creates strain for men, and leads to increased risky sexual behavior and conflict between partners [33–35]. Similarly, lack of economic opportunities can lead to feelings of hopelessness and distress that may cause interpersonal conflict for men [36]. Despite the detrimental effects of unemployment on men’s health, men have been overlooked in some poverty alleviation efforts, such as microfinance.

Microfinance extends small loans to poor individuals, often within groups, to start or support small business enterprise. A growing body of literature shows that beyond poverty alleviation, microfinance programs can lead to positive health outcomes [37–39]. The most striking example of positive health effects resulting from a combined microfinance and health intervention is from the Intervention for Microfinance and Gender Equity (IMAGE) study in South Africa. Women in this trial who received small loans and attended biweekly health sessions reported significantly less IPV, greater uptake of HIV counseling and testing, and greater HIV-related communication with partners than women in the control arm [39]. Women are generally the beneficiaries of microfinance because they have historically demonstrated better overall repayment and greater investment of these resources and profits in household needs [40, 41]. However, a growing number of microfinance organizations are committed to providing loans to men [42] and research has demonstrated positive health effects of microfinance with men, including greater use of contraception and reduced depressive symptoms among men in a South African study [43, 44].

We conducted formative research in Dar es Salaam to identify the venues where young men engaging in high-risk sexual behavior socialize. Through this formative research we identified camps, which we have described in previous publications [10, 45]. Camps are social networks of mostly men, and were found to have a median of 22 members and existed for an average of 8 years (range 4–13 years). Membership of camps is, on average, 80 % male. Most camps do not have offices or other permanent meeting rooms, but rather use public spaces, such as the side of a building to mark their meeting space. Camps have elected leadership and some require membership fees to belong. Most members belong to one camp and come every day for several hours to socialize. Camp members support one another in tangible ways such as contributing funds when members are ill. Male camp members also reported engaging in health risk behaviors at higher rates than those reported in the general population. For example, the six month cumulative prevalence of concurrency among sexually experienced male camp members was 42 % [10], substantially higher than other studies of male youth in sub-Saharan Africa that have found rates of concurrency that range between 20 and 38 % over time periods of past 12 months and past 3 years [46–50]. Our group has leveraged what we know about social networks of men to implement and evaluate a microfinance and peer health leadership intervention among social networks of young men in Dar es Salaam, Tanzania designed to reduce HIV risk behaviors and prevent IPV perpetration.

Social networks serve as an important context for shared peer norms related to HIV and provide opportunities for social influence processes to occur naturally [51]. Network-based interventions have the potential to transform these shared norms, leading to sustained behavior change. Microfinance combined with health leadership training may reduce STI incidence and the perpetration of violence by addressing the structural determinants and gender norms at the root of these issues. The health leadership intervention in particular, is designed to transform men’s attitudes towards gender roles to make them more equitable. In addition to transforming individual men’s attitudes, this intervention is designed to change camp-level norms as well. Additionally, the microfinance intervention is hypothesized to increase men’s hope for, and orientation towards the future. The combined approach is also meant to increase men’s perceived social support. Finally, this approach holds promise with regards to reducing risky behavior and violence in part because it is delivered at the camp level and the microfinance model relies on group members holding one another accountable for the repayment of their loans. As such, this intervention capitalizes on the structure and cohesiveness of peer groups or networks of individuals to keep each other motivated and to reinforce intervention messages. We anticipate that the combined intervention may work, in part, by making these camp networks more cohesive. Interventions designed not only to change the behavior of individuals but also to work with social networks to promote behavior change are likely to result in sustained change because they tap into naturally existing structures [51–53].

We conducted a pilot study to determine the feasibility and acceptability of a combined microfinance and peer health leadership training program for these social networks of young men in camps. We demonstrated that this innovative HIV and IPV prevention intervention was both feasible and acceptable, and results of the pilot study have been published elsewhere [54]. The lessons learned from this pilot have informed the implementation of the current cluster-randomized trial of the microfinance and peer health leadership intervention. This is the first cluster-randomized trial targeting social networks of men in sub-Saharan Africa that jointly addresses HIV and IPV perpetration and has biological and behavioral endpoints.

## Methods/Design

### Research design and methods

#### Overview

Vijana Vijiweni II is a cluster-randomized controlled trial, with the unit of randomization being camps. The trial tests whether men in camps randomized to a combined microfinance and peer health leadership intervention have less incident sexually transmitted infections (Neisseria gonorrhea (NG), Chlamydia trachomatis (CT), and Trichomonas vaginalis (TV) and report less past-year perpetration of physical and/or sexual violence against female sexual partners compared to men in the control camps who will get a delayed intervention. Sixty camps are enrolled in the trial and half are randomly assigned to the intervention arm and half to the control arm. All members who meet the study eligibility criteria (detailed below) are offered enrollment in the trial. Behavioral and biological data are collected at baseline, which is completed two months prior to initiating the intervention, and endline which occurs 30 months post intervention launch. We are also conducting a midline behavioral assessment at 12-months post intervention launch. The intervention runs for a period of 2 years.

##### Primary hypothesis

The combined microfinance and health leadership intervention will be associated with lower incidence of sexually transmitted infections and less perpetration of physical and sexual violence against female sexual partners among men in camps randomized to receive the intervention as compared to men in camps randomized to receive the control condition.

##### Secondary hypothesis

We hypothesize that several constructs will mediate the intervention effects. Specifically, at the individual level, men’s equitable gender role attitudes, hope, future orientation, and their perceived social support will mediate intervention effects. Additionally, at the camp level, the social cohesion as well as the structural cohesion of camp networks will mediate the effect of the intervention on the primary outcomes. We will also examine the intervention effects on secondary outcomes including unprotected sex, sexual partner concurrency, alcohol and other substance use.

### Setting

Dar es Salaam, the commercial capital of Tanzania, is one of the regions in Tanzania with the highest prevalence of HIV (6.9 %) [55]. Dar es Salaam is one of the fastest growing urban areas globally [56] and as a result the city has the highest population density (3,133 per sq. km), highest concentration of youth (13.4 % ages 15–24), and the highest unemployment rate (13 %) in the country [57]. Seventy-percent of city residents live in informal settlements [58]. Administratively, the city (1,393 sq. km) is divided into three districts, and 73 wards comprise these districts. Our study takes place in four wards within Kinondoni District. The population in these four wards range between 50,560 and 85,735 [59].

### Research ethics and approval and community engagement

This study is a collaborative effort between the University of North Carolina at Chapel Hill (UNC) and the Muhimbili University of Health and Allied Sciences (MUHAS) in Dar es Salaam. The IRBs at UNC and MUHAS approved the study and oversee adherence to the protocol. A Community Advisory Board (CAB) was established to liaise between research staff and the communities within which our study operates. Our CAB includes leaders of camps in our study, parents of participants, and local government authorities. CAB meetings are held at least once every 6 months to provide an update on the study and promote participant retention, and additional meetings are arranged if major study-related issues that need input from the CAB occur.

### Research activities and procedures

#### PLACE assessment to identify camps

**Fig. 1:**
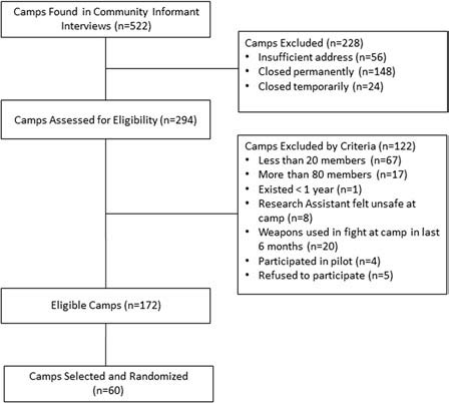
Consort diagram for camp selection

**Fig. 2:**
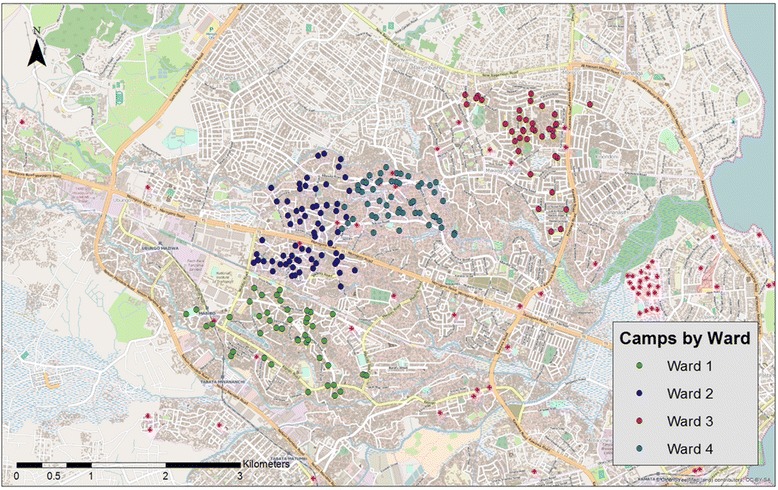
Map of camps by ward

We used the Priorities for Local AIDS Control Efforts (PLACE) method to identify, map, and characterize all camps in four wards of Dar es Salaam. PLACE is a venue-based sampling methodology that was developed as a surveillance tool for high-transmission venues [60, 61]. The PLACE assessment involves two steps to identify and verify the venues, including community informant interviews and venue-verification interviews. (1) *Community Informant Interviews:* We identified community informants in each ward who are familiar with the camps in their wards. We went *mtaa* by *mtaa* (street by street) and surveyed 489 individuals (432 men and 57 women) from the estimated 240,000 residents living in these wards. Those interviewed were asked to provide the following information on up to 10 camps within 10 min’ walk from where the interview was conducted: the camp name(s), the closest street name, and walking directions. Through the community informant interviews we identified 522 camps. (2) *Camp Verification Interviews:* Research assistants subsequently used directions collected during community informant interviews to verify camp existence and operation. Camps were omitted from the sampling frame if the directions were insufficient (*n* = 56) or if they had closed permanently (*n* = 148) or closed temporarily (*n* = 24). Of the 522 possible camps, 294 camps were included in the sampling frame and assessed for eligibility (Fig. 1). Geographical coordinates were collected for the remaining 294 camps and used to create a map of camp locations, which was constructed using ArcGIS 10.1 [62] (Fig. 2). We then approached all 294 camps to assess their eligibility by conducting a structured interview with the chairperson or another leader if the chairperson was unavailable. We conducted most interviews with the chairperson (*n* = 136), secretary (*n* = 54), or treasurer (*n* = 25). In the event none of the elected leaders were available, other camp members (*n* = 79) completed the interviews. Of the 294 camps assessed for eligibility, 172 were found to be eligible. Specifically, in order to be eligible for inclusion in our trial, camps had to have more than 20 members and less than 80 members (*n* = 67 and *n* = 17 camps were not eligible for these reasons, respectively). Camps also had to have been in existence for at least 1 year (*n* = 1 camp was ineligible as a result). Camps in which Research Assistants felt unsafe (*n* = 8) and camps in which a weapon had been used in a fight in the last 6 months (*n* = 20) were also excluded. Finally, camps could not have participated in pilot studies with our team (*n* = 4 camps were not eligible). An additional 5 camps refused to participate in the study.

#### Selection and random assignment of camps for the trial

Due to the density of geographically proximal camps (see Fig. 2), we used a three step, probability based sampling method that reduced the possibility of contamination effects to randomly select 60 eligible camps in to the trial: (1) field staff familiar with the physical geography of wards and camps assigned geographically proximal camps to groups containing between 1–6 camps on ArcGIS generated maps; (2) probability proportionate to size with minimal replacement sampling of groups was conducted using SAS Survey Select Procedure; (3) simple random selection of camps within groups selected was completed. By using minimal replacement, groups containing greater numbers of camps with high membership numbers could be selected more than once. In such an instance, the number of times a group was selected equaled the number of camps selected randomly from that group. The 60 randomly selected camps were first randomized into two sets of 30 camps. Multiple camps selected from within the same geographically proximally group were assigned to the same set. Treatment condition of these two sets was then randomly, and transparently, assigned at a CAB meeting following completion of baseline data collection. At the CAB meeting, the two sets were assigned numbers one and two. Papers with these numbers were then put in separate but identical balloons, and three other papers that stated “try again” in three other identical baloons. The CAB decided that the first number to be selected would be assigned to the intervention condition. One CAB member was selected to be the one to randomly pick and pop the balloons until a number was found.

#### Selection of participants from each study camp

**Fig. 3:**
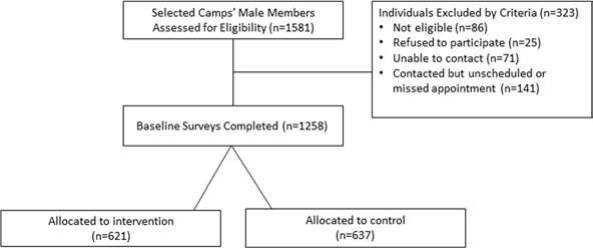
Consort diagram for individual participation

Following random assignment, a camp leader was given a camp roster to complete. The roster included information on each member of their camp (including their first and last name, nickname, birthdate, gender, phone numbers, length of time as camp member). We then contacted and scheduled interviews with all camp members. Prior to camp member enrollment, the team confirmed individual eligibility. In order to be eligible for participation in our trial, participants had to be older than 15 years, have been a camp member for more than 3 months, visit the camp at least once a week, plan on residing in Dar es Salaam for the next 30 months, and be willing to provide contact information for a friend or family member to be used in the event we could not contact the participant for future follow-up assessment. Of the 1,581 participants assessed for eligibility, 86 individuals (5.4 %) were confirmed to be ineligible and 25 (1.6 %) refused to participate. We reached but were unable to schedule appointments with 141 participants (8.9 %) and were unable to contact 71 individuals (4.5 %). There were no significant differences between the proportions of participants that were not contacted, ineligible, or refused to participate between the intervention and control arms. See Fig. 3 for Consort diagram of individual participation.

### Study assessments

Study assessments are conducted at baseline, 12-months post-intervention launch and at 30-months post-intervention. Behavioral surveys are collected at each assessment, and biological samples are collected at baseline and 30-month post-intervention only. Prior to the first assessment participants are asked for consent to participate in the study, which the participant provides by signing a consent form and writing their initials next to the assessments (behavioral, biological) to which they agree to participate. Participants must re-assent to participate in future behavioral assessments and consent to participate in biological assessment if not done at baseline. Behavioral surveys are conducted in field offices located in the study wards through computer-assisted personal interviews (CAPI) using tablet devices. Each interview is expected to take approximately 60–90 min to complete. Sixteen local interviewers, trained in human ethics, confidentiality, interviewing techniques, data collection methods, and use of CAPI devices, collect all behavioral data. To collect biological specimens, blood and urine samples are drawn from participants immediately after the behavioral survey is completed. To assess baseline prevalence of sexually transmitted infections (STIs), a subsample of 50 % of men that report ever having sex on their baseline survey are asked to provide a blood and urine specimen for HIV and STI tests. At the 30-month assessment, all men are asked to provide biological samples. Participants are refunded the equivalent of $3 for transport.

### Measures

The primary endpoints to assess intervention impact are STI incidence and IPV perpetration. To assess STI incidence, we are using Qiagen Artus CT with in-house PCR to detect CT, Trichomonas vaginalis Real-TM test to detect TV, and 16 s rDNA PCR to detect NG. For the follow-up testing at 30 months we are using Multiplex PCR to detect CT and NG. Study participants who are identified with NG, CT, or TV are offered free treatment by a study clinician at the study site according to the standard of care in Tanzania specifically NG (Ceftriaxone), CT (Doxycycline), TV (Metronidazole). IPV perpetration is assessed using an adapted version of the World Health Organization violence against women instrument [63]. This tool measures psychological, physical, and sexual IPV perpetration. Participants are asked whether they have perpetrated any of 13 behaviorally specific violent acts, including 4 psychological acts, 4 physical acts and 3 sexually violent acts, against a current or former sexual partner. For those who say yes to ever having perpetrated a specific act of violence, they are asked to report how many times they perpetrated that act in the last 12 months. Response options included never, once, 2–3 times, 4–10 times, and more than 10 times. The responses to the frequency of physical and sexual IPV perpetration within the last 12 months are dichotomized such that a 0 represents no sexual/physical IPV perpetration and a 1 represents at least 1 act of sexual or physical IPV perpetration within the last 12 months. We are measuring gender norms with the gender-equitable men’s scale [36], as well as hope [64], future orientation [65], and perceived social support as mediators of the intervention’s effects. We are measuring camp-level social cohesion using five items adapted from a measure of social cohesion developed by Sampson, Raudenbush & Earls [66]. For example, participants are asked how strongly they agree with statements like “people in my camp are willing to help each other” and “the members of my camp share the same values.” In addition, we are collecting data on all social ties within each camp at multiple waves of data collection. We are using these complete network data to assess whether our intervention is mediated through the transformation of social network structures (e.g. structural cohesion [67]). Finally, we are also looking at HIV risk behaviors as secondary endpoints including unprotected sex, sexual partner concurrency, alcohol and other substance use.

### Retention

Young men in this urban context are highly mobile, often moving for work-related opportunities. A number of strategies are employed to maximize participant retention through the life of the study. (1) Camp rosters containing member names and phone numbers were collected before the baseline assessment, and are updated with camp leader assistance before follow up data collection. (2) Phone numbers of participant family members and close friends are collected at baseline to assist in locating camp members difficult to track down at follow-up. (3) Bi-annual CAB meetings are held to maintain connection with camp leadership and check-in on camp membership. (4) For any respondent who leaves the camp and/or moves, we are using additional strategies such as finding out if they are still living around Dar es Salaam to contact them and encourage them to return for interviews, including providing additional funds for transport if they are located far from the field offices within Dar es Salaam.

### Qualitative sub-studies

We are conducting qualitative sub-studies during the study to follow-up in more depth on a few issues of particular interest to our study team. Our first qualitative study is focused on the *role of camps in men’s perpetration of violence.* This qualitative study is designed to understand more about the role that social networks within camps play in influencing men’s perpetration of violence against their female partners. In this sub-study, 20 men who report at least one episode of violence with their sexual partner in the past 12 months are selected for interviews. The interviewers ask men about how members of their camp talk to each other about relationships, and conflicts within relationships. They also ask for more information about the conflicts that selected participants have had with their partners to try to understand how they talked to camp members about these relationship conflicts. We conduct two interviews with each man. The second qualitative sub-study is focused on *camp leadership and microfinance uptake.* As we have implemented the intervention we have become increasingly aware of the important role that some camp leaders play in the functioning and decisions that are made within the camps. We will select 3 camps that took loans and 3 camps that did not and conducted semi-structured interviews with 2 leaders and 3-4 members per camp for a total of 30-36 interviews. We want to understand more about the strategies that camp leaders use to lead their camps, and how influential camp leaders are in the decisions of camps members regarding the uptake of loans. All qualitative interviews are conducted in Swahili by trained qualitative interviewers, audio recorded, transcribed, and translated for data analysis.

### Sample size determination

The choice of 60 camps randomized into two groups is based upon evidence generated in our previous study and likely incidence estimates available from published studies with similar populations in Tanzania and other countries in Africa [10, 68]. We estimated average camp sizes of 26.4 men and expected to successfully enroll approximately 80 % of these men into our trial at baseline. 90 % of the men were expected to be sexually active at baseline. Any STI incidence (NG, CT, or TV) was used as the primary outcome measure and sexual and/or physical IPV perpetration was used as the primary behavioral outcome measures. Based upon our previous study we computed camp intraclass correlation (ICC) estimates between 0.00 and 0.01 for sexually transmitted diseases and 0.032 for behavioral measures. Given anticipated average camp sizes and ICC estimates we inflated sample size estimates assuming simple random sampling with design effects ranging from 1.4 to 2.3. All sample estimates assumed an attrition of 20 %. Given a sample size of 890 sexually active males and 999 total males in 60 camps at our 30-month assessment, we will have 80 % power (2-sided, α = 0.05) to detect protective effects of the intervention (OR) of .57 for STI incidence and .65 for perpetration of sexual or physical IPV among sexually active men. Statistical power to detect mediation is expected to be high based on the simulation results of Fritz and MacKinnon where a total sample size of 539 was sufficient for .80 power to detect the most conservative simulated mediation effect.

### Data analysis

For our primary outcome analysis, we compare odds ratios for STI incidence and IPV perpetration in the past 12 months, across arms at 30 months for both behavioral and STI outcomes using SAS SURVEYLOGISTIC version 9.4 [69] to adjust for correlation due to clustering within camps and incorporate both the complex nature of sample selection and weighting. Controls for covariates will be introduced where necessary. In the event that program effects are identified, we will assess the degree to which hope, future orientation, and structural characteristics of the networks mediate the impact of the intervention on STI and IPV. Indirect (mediated) effects of the intervention will be calculated using Mplus and assessed for significance using construction of Monte Carlo confidence intervals [70].

### Intervention design and methods

The intervention involves two components, a microfinance component and a camp health leadership component. The intervention is implemented for a period of two years. We describe the design and methods for each component below.

### Microfinance component

**Fig. 4:**
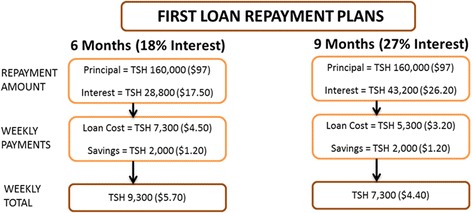
Diagram of weekly repayment plans

We partnered with a local microfinance organization, YOSEFO (Youth Self Employment Foundation) based in Dar es Salaam to implement the microfinance activities in this trial. All men in the camps randomized to the intervention arm who completed a baseline survey are eligible to participate. The intervention was developed based on standard microfinance practices. (1) *Business skills training:* Men who were eligible and interested in participating in the microfinance component are invited to a one week training facilitated by YOSEFO. Standard business/entrepreneurship training lessons include: how to generate viable business ideas; how to assess markets; how to start and scale up businesses; the business environment; how to manage businesses; entrepreneurship and the entrepreneur; marketing goods; cost, sales and pricing; customer service; effects of HIV/AIDS on ability to conduct business; and family and business. Finance lessons include: obtaining business capital; loan criteria; loan collateral; loan interest rates; loan groups and centers; loan application; and loan term and repayment. A key part of this initial training is teaching the participants about microfinance and the conditions of microfinance loans, so that they can make informed choices about their participation. *(2) Formation of loan groups:* Camp members are asked to form groups with four other camp members for the purpose of receiving the loan. Each camp member within the group receives an individual loan. While loans are paid to individuals, the groups are accountable for repayment of all group members’ loans *(3) Applications for loans:* Individuals apply for a loan by completing a loan application that includes details on the planned business, information on group members, listing of physical collateral against the loan, and evidence that they have U.S. $6 (TZS 10,0000) in savings. The loan application process occurs over a 3 week period and includes multiple steps, designed in part to get borrowers in the habit of attending meetings and depositing money. Loan applications are reviewed and approved by other group members, camp leaders and YOSEFO staff. (*4) Distribution of the loans.* After successful completion of the application, camp members receive an initial loan of $100 USD (THS 160,000) at 18 % interest on a 6- month repayment cycle or 27 % on a 9-month repayment cycle. (5) *Weekly repayment sessions:* The camp members meet with a loan officer in their ward on a weekly basis with their group to repay their loan principal plus interest. Loan access and repayment data is entered into an Access database by Loan Officers at these weekly sessions. Weekly repayment amounts are determined by dividing the loan plus interest by the number of weeks in their loan term. Borrowers are also expected to deposit TZS 2,000 (U.S. $1.25) in savings at each repayment session. All members are expected to attend these repayment sessions. Group members are responsible for ensuring payment from all members of their group at weekly repayment sessions. See Fig. 4 for a diagram of the weekly repayment plan for men on the 6- and 9-month cycles. Should one group member fail to pay, it is the responsibility of other members to cover the payment. In the event that an individual defaults on a loan, all members of the particular group with loans are responsible for repayment of the outstanding loan balance. Men failing to repay their loans within the loan term are approached to secure the collateral listed on their loan application until loan repayment is complete. *(6) Ongoing access to credit*: Since ongoing access to incrementally larger loans is a key feature of microfinance, camp members completing repayment of the first loan are eligible for a second loan in the amount of $185 USD (TZS 300,000), and if successful with that a third loan in the amount of $280 USD (TZS 450,000). An agreement with YOSEFO was established at the outset such that any camp member who successfully completes three loan cycles during the duration of this study, will be eligible to apply for additional loans through YOSEFO following standard practices for the organization*. (7) Monitoring MFI repayments*: The study team monitors all loan disbursements, weekly amounts repaid versus expected, savings deposited, and portfolio at-risk. Data are entered to an Access client database in Tanzania and synced weekly to a web based server. Aggregated data and pre-configured statistics are accessed through a master database in the U.S. Graphs of repayment success are created bi-monthly by the study team to monitor progress. This strategy allows us to identify patterns and problems early so that we can isolate the problems and try to develop strategies to address problems as they arise.

### Peer Health Leader component

The second component of the intervention targets peer leaders within camps, called camp health leaders (CHL), and trains them as health promoters for behavior change. This intervention component builds on the fact that it is possible to identify peer nominated leaders within the camps who are respected and trusted by their peers. We use the following steps to implement this component: *(1) Nomination of leaders:* Based on recommendations of previous studies [71] that have used peer opinion leaders to promote health behavior change in networks we aim to recruit 20 % of all camp members as CHLs. To identify CHLs we hold meetings with camp members in each camp. Prior to a nomination, members are asked to describe attributes of a leader. Research staff note these attributes and supplement the list with pre-determined qualities wanted in CHLs (e.g. someone who could be trusted with personal matters, someone whom you admire). At the conclusion of this discussion each camp member confidentially nominates up to 3 leaders in their camps with the qualities documented. Votes are tallied by the research coordinator. The top 20 % of camp members receiving the most votes in each camp are approached and asked if they are interested in participating in the camp health leader training. *(2) Training:* Peer leaders participate in an initial one-week training and two, single day booster training sessions held two and five months later. The trainings are facilitated by the site-PI and the Project Coordinator who are both master’s trained psychologists with expertise in training for counseling in this context. The training provides the leaders with knowledge to address myths and misconceptions related to HIV transmission and prevention, condoms, violence and multiple sexual partnerships. A major component of the training is building skills in effective communication for social influence. Through role playing and demonstrations, leaders learn how to engage their peers in conversations about sensitive topics, how to identify and address barriers to practicing safe sex, how to counter negative viewpoints, how to use ‘I’ statements when talking with their peers about behavior, how to be better listeners, and how to model positive behavior choices for their peers. The training module on gender-based violence includes several interactive activities that are designed to help the peer leaders clarify their own attitudes and values related to gender, violence and power and to help the leaders understand how violence affects women’s health and well-being. Additional booster training sessions are held bi-annually with the CHLs to review information covered in the initial training, and discuss success and challenges that the health leaders faced in implementing the strategies among their peers. CHLs receive a modest allowance (TZS 5,000, equivalent to USD 2.44) for the training days to cover costs associated with transportation to get to and from the training venue. *(3) Implementation of the communication and social influence strategies:* Once the CHL leaders are trained, they are asked to implement the strategies they learned among peers in the camps. This includes incorporating the material they learned in the training into naturally occurring conversations that they are having with members of their camp. *(4) Monitoring CHL conversations*: CHL are required to keep a weekly diary documenting the number of HIV and GBV specific conversations they have had with other camp members. We established a goal with the CHL of 5 conversations per week within the camp, to try to monitor dosage of conversations against this goal. These diaries are collected from CHLs and entered into an Access spreadsheet in Tanzania and synced weekly to a web-based server. Aggregated data and pre-configured statistics are accessed through a master database in the U.S. Graphs of the conversations by camp are created and viewed bi-monthly to identify CHLs who are achieving and those who are not achieving our target dosage goals.

## Discussion

This is the first intervention trial that we know of targeting social networks of men in sub-Saharan Africa that jointly addresses HIV and IPV perpetration and will be evaluated with behavioral and biological endpoints. Our team identified social networks of mostly men who socialize in venues called camps. We are leveraging what we learned about these camp-based social networks to develop, implement and evaluate an intervention that combines microfinance with health promotion training for network leaders. The intervention is being evaluated through a cluster-randomized trial, in which 60 camps have been randomly selected across 4 wards of Dar es Salaam, and then randomized to receive the combined intervention or the control condition.

The study has a number of strengths. First, we have overcome challenges associated with identifying and reaching social networks of young men at risk for HIV and IPV perpetration. We have leveraged what we learned about these camp-based peer networks to design and implement the intervention trial. Second, we have built on intervention approaches that have demonstrated efficacy in leading to behavior change in other populations. We expanded what we know about these intervention approaches and applied it to reach young men in Dar es Salaam. We demonstrated that the application of these intervention approaches is feasible and acceptable through a pilot study prior to the implementation of the trial. Third, the intervention is being rigorously evaluated using a cluster-randomized trial design. Finally, we are carefully monitoring the intervention implementation through a process evaluation system that is designed to provide feedback and allow for midcourse improvements to enhance implementation.

The study also has limitations. While the camps are generally stable networks of men, it’s possible that men’s engagement in camps may vary over time, and camp membership may shift over time, making it challenging to retain men in the trial. Second, as with all cluster-randomized trials, there is the potential for contamination, and particularly in our case since we are working in a densely population urban setting. We were careful to group contiguous camps to minimize contamination across camps that are most proximal, however, it is still possible given the dense urban setting, that camp members in camps assigned to different conditions may share information. To measure the magnitude of contamination, intervention exposure is measured in both intervention and control camps during the 12-month and 30-month survey by asking participants to identify the other camps that they visited and assessing the frequency with which they visit these camps. We also ask participants whether they talk to individuals in those other camps about HIV or gender-based violence during the intervention period. Finally, it is possible that by working with existing social networks, characteristics of these networks such as how cohesive they are may influence how camp members do or do not engage in the intervention activities. As part of our secondary analysis, we examine how network characteristics influence the study outcomes across the camps. Our documentation of the network characteristics and their influence on study implementation and outcomes will contribute valuable information to the scientific understanding of how to design and implement social network interventions.

Effective approaches to engage men in HIV and IPV prevention are needed in low resource, high prevalence settings like Tanzania. If we determine that this approach is effective, we will examine how to adapt and scale up this approach to other urban, sub-Saharan African settings.

### Availability of data and materials

Data collected through this study and used in analyses will be made available as additional supporting files for main papers. At the conclusion of our main study analyses data will be made available upon request and in accordance with U.S. National Institute of Health regulations.
